# Regulation of the transforming growth factor β pathway by reversible ubiquitylation

**DOI:** 10.1098/rsob.120082

**Published:** 2012-05

**Authors:** Mazin A. Al-Salihi, Lina Herhaus, Gopal P. Sapkota

**Affiliations:** Medical Research Council—Protein Phosphorylation Unit, Sir James Black Centre, University of Dundee, Dow Street, Dundee DD1 5EH, UK

**Keywords:** transforming growth factor, ubiquitylation, ubiquitination, deubiquitylation, DUBs, ubiquitin

## Abstract

The transforming growth factor β (TGFβ) signalling pathway plays a central role during embryonic development and in adult tissue homeostasis. It regulates gene transcription through a signalling cascade from cell surface receptors to intracellular SMAD transcription factors and their nuclear cofactors. The extent, duration and potency of signalling in response to TGFβ cytokines are intricately regulated by complex biochemical processes. The corruption of these regulatory processes results in aberrant TGFβ signalling and leads to numerous human diseases, including cancer. Reversible ubiquitylation of pathway components is a key regulatory process that plays a critical role in ensuring a balanced response to TGFβ signals. Many studies have investigated the mechanisms by which various E3 ubiquitin ligases regulate the turnover and activity of TGFβ pathway components by ubiquitylation. Moreover, recent studies have shed new light into their regulation by deubiquitylating enzymes. In this report, we provide an overview of current understanding of the regulation of TGFβ signalling by E3 ubiquitin ligases and deubiquitylases.

## 2. The transforming growth factor β signalling pathway

The transforming growth factor β (TGFβ) family of cytokines control a plethora of cellular processes, including proliferation, differentiation, extra-cellular matrix production, motility and survival [1,2]. These translate into critical tissue functions throughout embryogenesis and adult life, achieved by striking a balance between proliferation and differentiation [2–4]. When this balance is perturbed, the TGFβ pathway malfunctions. Aberrant TGFβ signalling is associated with many human diseases including immune disorders, fibrosis, cancer progression and metastasis [5–12]. Therefore, understanding the molecular mechanisms underpinning the regulation of the TGFβ pathway would facilitate novel therapeutic opportunities against these diseases.

**Figure 1.**
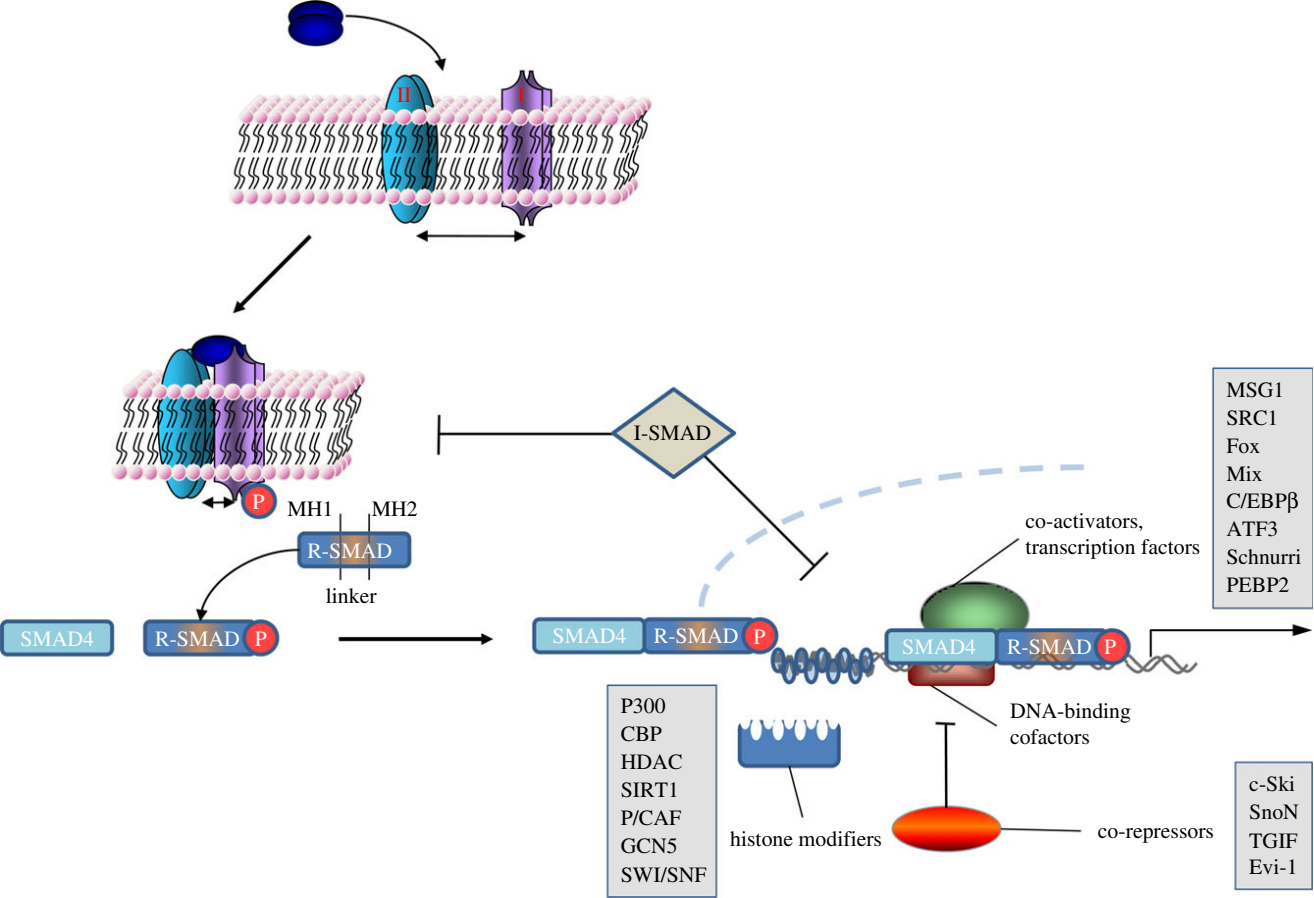
The logic of TGFβ signalling from the membrane to nucleus. Upon ligand binding, the TGFβ/BMP receptor kinases mediate the phosphorylation of R-SMADs. R-SMADs are depicted showing their MH1/Linker/MH2 domains. This induces the association of R-SMADs with SMAD4 and their nuclear translocation. In the nucleus, the SMADs form transcription complexes with multiple cofactors and regulate the transcription of multiple target genes. Most of the known transcriptional cofactors of SMADs are indicated, although not all are described in the text.

TGFβ signalling is initiated when ligands bind to their cognate receptors (figure 1). There are at least 42 different TGFβ ligands, which are divided into two main subgroups: the TGFβ family and the bone morphogenetic protein (BMP) family. Ligand binding induces specific quaternary complex formation of the transmembrane serine threonine kinase receptors. These receptors are divided into type I (ALK1-7) and type II (ACVR-IIA, ACVR-IIB, BMPR-II, AMHR-II and TGFβR-II). SMAD proteins are the intracellular transducers of the pathway; they are divided into specific subgroups: receptor-regulated (R-SMADs; 1–3, 5 and 8), the co-SMAD (4) and the inhibitory (I-) SMADs (6 and 7). Upon ligand binding, the type II receptors phosphorylate and activate the type I receptors. Activated type I receptors phosphorylate the R-SMADs at their C-terminal SXS motif. This induces R-SMAD complex formation with SMAD4 and nuclear translocation, where along with their nuclear cofactors they bind DNA and regulate transcription. The vast number of ligands and receptors allows for the formation of unique ligand–receptor complexes in distinct biological settings. In general, the TGFβ receptor subfamily signals through SMADs 2 and 3, while the BMP subfamily signals through SMADs 1, 5 and 8, although some crosstalk between the two pathways has been reported. A negative feedback loop is created by TGFβ- or BMP-induced transcription of the I-SMADs. I-SMADs inhibit the pathway by competing with R-SMADs for association with the type I receptors, or by recruiting E3 ubiquitin ligases and targeting the receptors for degradation. In the nucleus, a variety of nuclear cofactors are required for the R-SMADs to bind DNA and induce gene transcription (figure 1). Additionally, various histone and DNA modifiers are required for opening or closing sections of DNA to transcriptional regulation by R-SMADs [1,13–18]. While we focus on the role of reversible ubiquitylation in regulating the core components of the TGFβ pathway in this review, they can be further regulated by multiple post-translational modifications, which also impact the outcome of TGFβ signalling. Often it is the integration of all the regulatory inputs that determines the cellular responses to TGFβ signals.

## 3. Reversible ubiquitylation

Ubiquitylation, also referred to as ubiquitination, is a reversible process by which ubiquitins are attached to proteins, either singly or in chains. This post-translational modification causes target proteins to undergo changes in stability, subcellular localization or activity. Ubiquitin is a member of a conserved family of small eukaryotic proteins (approx. 8.5 kDa) that share the ubiquitin fold structure. Through an isopeptide bond, ubiquitin is attached to lysine residues on the target, creating mono-ubiquitylated proteins. Attaching more ubiquitin molecules to the lysines of an already protein-bound ubiquitin creates polyubiquitin chains. Depending on which lysine the subsequent ubiquitin molecules are attached to, different fates await the polyubiquitylated proteins. While eight linkage types are possible (through K6, K11, K27, K29, K33, K48, K63 and α-amino group of ubiquitin) as well as mixed chains, not all have been attributed a function. Two linkage types are commonly studied and reported in the TGFβ pathway: K48 chains are known to signal protein degradation, while K63 chains play a role in signalling as well as in protein trafficking and endocytosis.

Ubiquitin attachment is achieved through a three-step process catalysed by an E1-ubiquitin-activating enzyme, specific E2-ubiquitin-conjugating enzymes and a wide array of E3-ubiquitin ligases. E1 enzymes activate and transfer ubiquitin in an ATP-dependant manner to the E2-ubiquitin-conjugating enzyme. This high-energy ubiquitin–E2 conjugate then specifically interacts with the E3-ubiquitin ligase, which could be either a single protein or part of a larger ligase complex. E3s can be divided into three structural groups, U-Box, HECT and Ring E3s, depending on their conserved domains and mode of catalysis. Several ubiquitin-like proteins (UBLs), including SUMO1-3, NEDD8, FUBI, HUB1, ISG15, FAT10, URM1, UFM1, Atg12 and Atg8, share a similar three-step attachment process. However, these UBLs use different E1, E2 and E3 enzymes. While SUMO (small ubiquitin-like modifier) has been reported to modulate TGFβ signalling, this review will concentrate on ubiquitin [19–24].

The removal of ubiquitins or polyubiquitin chains from the target protein is catalysed by deubiquitylating enzymes (DUBs). Therefore, DUBs reverse the function of E3 ubiquitin ligases [25]. DUBs remove ubiquitin from cellular adducts, process inactive ubiquitin precursors, proofread ubiquitin–protein conjugates and protect the 26S proteasome from ubiquitin chain accumulation [26]. Furthermore, DUBs generate free ubiquitin by removing and chopping ubiquitin chains from proteins, leading to recycling of ubiquitin, thereby contributing to ubiquitin homeostasis. The fate of ubiquitylated proteins can be further modified by DUBs that edit or trim ubiquitin chains, resulting in a reversal of ubiquitin signalling. This could lead to protein stabilisation by rescue from degradation [25]. Deubiquitylation is implicated in several cellular functions such as gene expression, DNA repair, cell cycle regulation, kinase activation and microbial pathogenesis [27].

DUBs are classified into five distinct functional and structural groups: the zinc metalloproteases JAMM/MPN+, and the cysteine proteases, comprised of ubiquitin C-terminal hydrolases (UCHs), ubiquitin-specific proteases (USPs), ovarian tumour proteases (OTUs) and Josephins [25]. There are also DUBs that resemble the adenovirus protease that cleave interferon-stimulated gene 15 (ISG15) conjugates and ubiquitin-like proteases (ULPs), which belong to the Adenain family of cysteine proteases, that are specific to ubiquitin-like proteins SUMO or NEDD8 [27]. As the human genome encodes less than 100 DUBs, it is evident that DUBs are highly regulated and play a role in diverse signalling pathways in order to oppose the action of over 600 E3 ligases [25,28]. A combination of substrate and target choice determines overall DUB specificity, which is further regulated by conformational/post-translational changes, subcellular localization and interactions with cofactors. DUBs distinguish between ubiquitin-like molecules, isopeptides, linear peptides and different types of ubiquitin linkage and chain structures as well as *exo*- versus *endo*- deubiquitylation to ensure specificity. Enzymatic activity of DUBs is often cryptic and regulated by occluding the substrate-binding site of certain DUBs or by inducing conformational changes that activate the catalytic site. Apart from these substrate-induced conformational changes and post-translational covalent modifications, activity can also be regulated by interacting cofactors. Other events, such as transcriptional regulation of DUB expression and subcellular localization, further ensure specific ubiquitin chain cleavage. DUBs are modular and contain multiple domains that mediate protein–protein interactions, apart from their catalytic domains. These domains include ubiquitin-binding domains (UBDs) or ubiquitin-like folds (UBL folds), ubiquitin-interacting motifs (UIMs), zinc finger USP domains (ZnF-UBP domain) and ubiquitin-associated domains (UBA domains). These domains contribute to the binding and recognition of different ubiquitin chain linkages but some DUBs also display direct affinity for their ubiquitylated target protein [25,27,28]. Recent studies have demonstrated that DUBs play critical roles in the TGFβ pathway regulation [7,29,30]. However, the field requires further research in order to identify DUBs that regulate the TGFβ pathway and understand their mode of action. Understanding the precise roles of DUBs in regulating the TGFβ pathway may unravel new opportunities for therapeutic intervention.

## 4. Regulation of the TGF β pathway components by reversible ubiquitylation

The fundamental steps and the key players in the TGFβ pathway are generally well defined. In this review, we focus on our understanding of how reversible ubiquitylation impacts three groups of key TGFβ pathway mediators: the TGFβ receptors, the SMAD transcription factors and nuclear SMAD cofactors. By integrating multiple signals, reversible ubiquitylation of these components in different biological contexts plays crucial roles in balancing the outcome of TGFβ signalling. Defective ubiquitylation of the TGFβ pathway components has been implicated in many human diseases, especially cancer [7,8,11,12,31–34].

## 5. Reversible ubiquitylation of TGF β receptors

**Figure 2.**
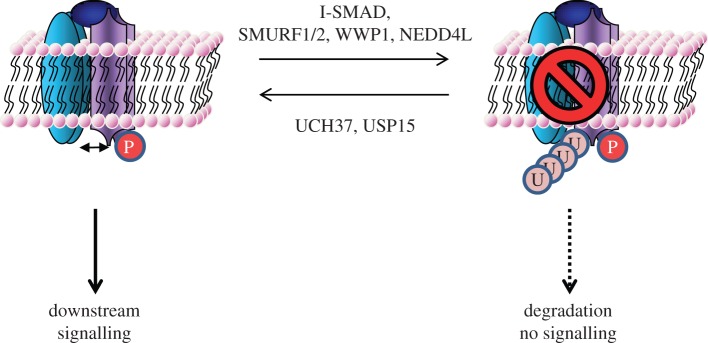
Regulation of the TGFβ–BMP receptor complexes by reversible ubiquitylation. Sketch of how reversible ubiquitylation of the receptor complexes may regulate pathway signalling. Detailed description is covered in the text.

Receptor complex assembly and activation upon binding TGFβ ligands are central to the activation of intracellular signalling. The activity and integrity of type II and type I TGFβ receptors can be modulated by several strategies: dephosphorylation of the activated receptors, interfering with the receptor/R-SMAD binding, changing receptor localization and/or targeting receptors for proteasomal degradation. I-SMADs play a crucial role in some of these strategies by modulating the activity and stability of active TGFβ receptor complexes. SMAD7 was reported to inhibit the TGFβ pathway by not only interfering with R-SMAD phosphorylation but also recruiting the E3 ubiquitin ligases SMURF1 and SMURF2 to the receptor complex (figure 2) [35,36]. This led to both receptors (ALK5 and TGFβR-II) and SMAD7 being ubiquitylated and targeted for degradation. Similarly, SMAD6/7 has been described to direct SMURF1 to ALK6 and mediates receptor ubiquitylation and degradation [37]. Both I-SMADs and SMURF1/2 are transcriptional targets of TGFβ and BMP signals, thereby creating a negative feedback loop [38,39]. A glycosyl phosphatidylinositol-anchored protein, CD109, further enhances the SMAD7–SMURF2 receptor complex interaction, strengthening the negative feedback [40,41]. Conversely, a recent study demonstrated that a protein named TGF-β-stimulated clone 22 (TSC-22), which is induced by TGFβ, inhibits the SMAD7–SMURF complex from binding, ubiquitylating and degrading the receptor complex. As expected, this leads to enhanced TGFβ signalling that translated physiologically into increased TGFβ-induced cellular differentiation [42]. Tribbles homologue 3 (TRB3) is another TGFβ-induced gene capable of enhancing pathway signalling in a positive feedback loop. TRB3 enhances SMAD3 nuclear localization and induces degradation of SMURF2 promoting cell migration, invasion and epithelial to mesenchymal transition (EMT) [43]. In human renal cell carcinomas, enhanced SMURF2 expression causes the reduction in levels of type II TGFβ receptor by proteasomal degradation [8]. SMURF1 and SMURF2 belong to the NEDD4-like family of HECT E3 ubiquitin ligases and are characterized by the presence of a conserved C2-WW-HECT domain structure [44]. While the C2 domain regulates the subcellular localization, the WW domains are 38–40 residue motifs characterized by two highly conserved tryptophans and folded as a three-strand β sheet that associate with the proline-rich ‘PPXY’ motif (also known as ‘PY’ motif) [45]. The PY motif present in the linker region of SMAD7 interacts with one of the WW domains of SMURF1/2 [35]. Other members of the NEDD4-like family, WWP1 and NEDD4L, have also been shown to interact with SMAD7 and target ALK5 for ubiquitylation and degradation. However, unlike SMURF1/2, they did not target SMAD7 itself for ubiquitin-mediated degradation, possibly providing a stronger and longer lasting negative regulation of the pathway [46–48]. In our studies, we have identified three further members of the NEDD4-like family of E3s, namely NEDD4, WWP2 and ITCH, as SMAD6/7 interactors. These are also likely to act in a similar mode to regulate the activity and stability of the TGFβ receptors. The precise nature of ubiquitin attachment and the sites for ubiquitylation on TGFβ receptors remain undefined. While several E3s have been implicated to act on the TGFβ receptors, to date very few E2-ubiquitin-conjugating enzymes have been assigned. SMAD7 has been reported to facilitate the recruitment of UbcH7, an E2 enzyme, to SMURF2 thereby providing a pathway-specific control on SMURF2 activity [49].

The regulation of TGFβ receptors by DUBs would be predicted to reverse the effects of E3 ubiquitin ligases and positively regulate the TGFβ pathway. To date, only two studies have addressed deubiquitylation of the receptors. UCH37 was reported to target ALK5 for deubiquitylation thereby enhancing TGFβ signalling [50]. However, structural studies on the UCH family of DUBs imply they are di- or polyubiquitin chain editors [25]. USP15 was identified as a modulator of TGFβ-induced transcription from a pan DUB-*siRNA* screen and subsequently shown to act on ALK5. The study further linked USP15 gene amplification with poor prognosis in glioblastoma [7].

## 6. Regulation of SMAD transcription factors by reversible ubiquitylation

**Figure 3.**
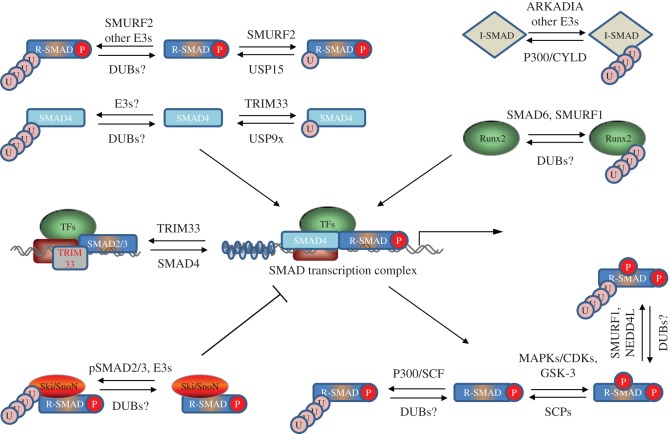
Regulation of SMAD transcription factors and nuclear cofactors by reversible ubiquitylation. An overview of how reversible ubiquitylation of SMAD transcription factors and associated nuclear cofactors may impact the SMAD-dependent transcription. Most of the reported E3s and DUBs known to regulate specific proteins are included. The reported mechanisms by which different E3 ubiquitin ligases and DUBs regulate SMAD proteins and associated cofactors are described in the text.

SMAD proteins are the intracellular transducers of TGFβ signals. R-SMADs are phosphorylated at their C-terminal SXS motif inducing complex formation with SMAD4 and nuclear translocation. In the nucleus, they induce transcriptional responses of TGFβ target genes. Interfering with R-SMAD phosphorylation, stability, R-SMAD/SMAD4 complex formation or DNA binding would negatively impact TGFβ pathway signalling. Reversible ubiquitylation of SMADs directly impacts one or more of these attributes. Here, we provide an overview of how reversible ubiquitylation of SMAD transcription factors impacts SMAD function and pathway signalling. Figure 3 summarizes the key players regulating reversible ubiquitylation of SMADs.

## 7. The BMP pathway SMADS

The first E3 ligase reported to ubiquitylate BMP-responsive SMADs was SMURF1 [51]. The WW domain of SMURF1 interacts with the PY motif of SMAD1/5 and targets them for ubiquitylation and proteasomal degradation [51,52]. Studies in *Xenopus* embryos showed that SMURF1 causes dorsalization of ventral mesoderm and neuralization of ectoderm, phenotypes consistent with inhibition of the BMP pathway [51]. SMURF1-mediated SMAD1/5 ubiquitylation promotes myogenic differentiation of C2C12 cells, blocks BMP-2-mediated osteogenic conversion [52] and modulates the effects of BMP4 on embryonic lung growth [53]. In contrast, SMURF1 has been shown to have little effect on TGFβ-inhibited myogenic differentiation [51,52]. LMP-1, an LIM domain protein capable of inducing de novo bone formation that contains a WW domain, interacts with SMURF1 and competes with SMAD1/5 for binding . Consequently, LMP-1 prevents SMURF1-mediated SMAD1/5 ubiquitylation and results in increased cellular responsiveness to BMP signals [54]. The PY motif in SMAD1/5 is preceded by a cluster of Ser/Thr residues. Phosphorylation of these residues, catalysed by proline-directed Ser/Thr protein kinases (e.g. MAP kinases and CDK8/9), in response to different stimuli as well as glycogen synthase kinase-3 (GSK-3) is essential for its interaction with SMURF1 [55–57]. BMP-induced sequential linker phosphorylation of SMAD1 by CDK8/9 and GSK-3 primes SMAD1 for transcriptional action and degradation, respectively. While phosphorylation by CDK8/9 induces recruitment of YAP1 mediator through its WW domain, subsequent phosphorylation by GSK-3 displaces YAP1 and recruits SMURF1 [45,55]. YAP1 stability is further regulated by SCF (Skp, Cullin, F-box)–βTRCP-induced ubiquitylation [58]. These studies demonstrate a clear interplay between phosphorylation and ubiquitylation in balancing the outcome of BMP pathway signalling. SMURF2 has also been shown to polyubiquitylate SMAD1 and mediates its degradation. Studies in *Xenopus* embryos confirmed that SMURF2 inhibits SMAD1 responses [59,60]. SMAD8 lacks the PY motif in its linker region and would be predicted to be resistant to SMURF-mediated ubiquitylation and degradation. A U-box-dependent E3 ubiquitin ligase member carboxyl terminus of Hsc70-interacting protein (CHIP) was reported as an interactor of SMAD1. CHIP was shown to cause ubiquitylation and degradation of SMAD1, resulting in the inhibition of the BMP-induced transcriptional activity [61]. The lysine residues within BMP–SMADs modified by ubiquitylation, the nature of polyubiquitin linkages and the E2-ubiquitin-conjugating enzymes involved remain to be defined. No DUBs for BMP–SMADs have been reported.

## 8. The TGF β pathway SMADs

Among the SMADs, TGFβ–SMAD ubiquitylation has received the most scrutiny. The evidence for polyubiquitylation and degradation of TGFβ-induced phospho-SMAD2 was first demonstrated in 1999 [62]. Subsequently, several E3 ubiquitin ligases, including SMURF1/2, NEDD4L and WWP1, have been implicated in mediating the polyubiquitylation and degradation of SMAD2/3 [47,48,59,63]. These NEDD4-like E3 ubiquitin ligase members all use the PY motif present in the SMAD2/3-linker for interaction. However, the recruitment of NEDD4L to SMAD2/3 requires the phosphorylation of the linker region mediated by CDK8/9 as well as the PY motif [64]. A WW-domain-containing protein PIN1 has been implicated in recruiting SMURF2 to linker-phosphorylated SMADs [65]. NEDD4L itself is also subject to further regulation by serum/glucocorticoid-regulated kinase 1 (SGK1), which is itself a transcriptional target of TGFβ signalling [64]. Signal termination is also achieved by other E3 ligases, independent of linker phosphorylation, using SMAD2/3 interactions with transcriptional cofactors. The ROC1–SCF–βTRCP RING E3 ligase complex targets activated SMAD3 for nuclear export and ubiquitin-mediated degradation upon its association with the transcriptional co-activator p300 [66] . The transcriptional regulator TAZ, reported to be required for SMAD2/3/4 complex nuclear accumulation, is also regulated by SCF–βTRCP-induced ubiquitylation and degradation [67,68]. While the previous examples show SMAD2/3 regulation after TGFβ signal initiation, CHIP has been shown to interact with ubiquitylate and degrade basal SMAD3 levels, resulting in the inhibition of TGFβ signalling [69].

SMURF2 features prominently in reports describing SMAD2/3 degradation. SMURF2-mediated inhibition of TGFβ signalling has been demonstrated across multiple organisms and in obstructive nephropathy [34,39,65,70]. One area of contention is whether SMURF2 polyubiquitylates [39,65,70] or monoubiquitylates [34] the TGFβ SMADs, targeting them for degradation or inhibiting complex formation with SMAD4, respectively. Nonetheless, both outcomes are reported to result in TGFβ signalling inhibition.

A member of the RING E3 ubiquitin ligase family, ARKADIA, was initially reported to ubiquitylate phosphorylated SMAD2/3 in the nucleus [71]. Despite this, ARKADIA resulted in enhanced TGFβ signalling. While counterintuitive, this was consistent with previous reports showing the effect of ARKADIA on Nodal signalling [72,73]. It was later shown that ARKADIA targets the inactive phospho-SMAD2/3-SKI complex for ubiquitylation and degradation [74]. SKI is a nuclear cofactor that negatively regulates TGFβ signalling by binding phosphorylated SMADs and preventing their transcriptional activity. ARKADIA balances SKI and SMAD2/3 ubiquitin-mediated degradation enhancing pathway transcriptional responses while terminating signalling once that is achieved. Changing the balance leads to TGFβ-pathway-related pathology in colorectal cancer where mutations leading to a reduction in ARKADIA function have been reported [11]. WWP1/2 have also been implicated in ubiquitylation and destabilization of SMAD2/3 [48,75]. While ITCH and CBLB E3 ubiquitin ligases have been reported to ubiquitylate SMAD2, they promote TGFβ-induced SMAD2 phosphorylation and signalling [76–78] . Although ITCH mediates the attachment of K48-linked ubiquitin chains on SMAD2, no degradation is observed [77]. This indicates that certain K48-linked polyubiquitin chains may have functions beyond proteasome-mediated degradation.

Despite numerous E3 ubiquitin ligases proposed to ubiquitylate the TGFβ SMADs, USP15 is the only deubiquitylase reported to act on SMAD2/3 [30]. USP15 has been reported to reverse SMAD2/3 monoubiquitylation, which targets the DNA-binding domains of SMAD2/3 and inhibits promoter recognition. The DUBs reversing the polyubiquitylation of SMAD2/3 remain to be defined.

## 9. SMAD4

Association of SMAD4 with R-SMADs is a critical step in the canonical TGFβ and BMP signalling pathways. Preventing this association or targeting SMAD4 for degradation inhibits TGFβ/BMP signalling. Regulation of SMAD4 by both mono- and polyubiquitylation has been reported [61,79–84]. Despite the lack of an intact PY motif, SMAD4 is polyubiquitylated by SMURF1/2, WWP1 and NEDD4L, which are recruited to SMAD4 by their association with I-SMADs and SMAD2 [83]. The E3 ligase CHIP has been implicated in controlling SMAD4 stability; however its role in SMAD4 ubiquitylation is unclear [61]. SCF complexes have been reported to ubiquitylate and degrade SMAD4. β-TRCP1 was initially shown to bind SMAD4 and induce its ubiquitin-mediated degradation through SCF. In the absence of SMAD4, the over-expressed complex was unable to inhibit TGFβ-induced cell cycle arrest [84]. SCF–βTRCP1 complex has been reported to control SMAD4 stability in pancreatic cancer cells [12]. The other SCF complex with SKP2 was also shown to bind and degrade SMAD4 [82]. Interestingly, TGFβ induces destruction of SKP2 in the nucleus, providing a further layer of control in the feedback loop [81].

The RING E3 ubiquitin ligase TRIM33 (also known as Ectodermin/TIF1γ), which also contains a plant homeodomain (PHD)—Bromo domain, has been proposed to interact with and ubiquitylate SMAD4 [80]. Although the critical role of TRIM33 on the TGFβ pathway is not debated, reports on the mechanisms by which it achieves this differ greatly. Two modes of action have been proposed. (i) TRIM33 interacts with phosphorylated SMAD2/3 in competition with SMAD4, thereby interfering with SMAD2/3–SMAD4 binding and creating separate SMAD2/3–SMAD4 and SMAD2/3–TRIM33 complexes, each resulting in distinct functions on cellular processes [85,86]. Furthermore, the PHD-Bromo domain has been demonstrated to be essential for the recruitment of TRIM33 to chromatin [79,86]. (ii) TRIM33 directly interacts with SMAD4 and not SMAD2/3, catalyses its polyubiquitylation [80] or mono-ubiquitylation at Lys519, which inhibits SMAD2/3–SMAD4 complex formation [29]. It has been shown that chromatin binding is required for the E3 ligase activity of TRIM33 *in vitro* [79]. While targeted disruption of the TRIM33 gene in mice has established the role for TRIM33 in limiting Nodal responsiveness *in vivo* [87], it has not resolved the debate on its mode of action. A mouse or a cell-line model in which wild-type TRIM33 is replaced by a catalytically inactive mutant with an intact PHD-Bromo domain would resolve definitively the issue of whether the E3 ligase activity of TRIM33 on SMAD4 is necessary for its influence on the TGFβ pathway.

USP9X/FAM is the only deubiquitylase reported to reverse the monoubiquitylation of SMAD4 at Lys519 mediated by TRIM33 [29]. Depletion of USP9X resulted in inhibition of TGFβ-induced transcriptional and cellular responses but not phospho-SMAD3. USP9X interacted with and deubiquitylated SMAD4 [29].

## 10. Inhibitory SMADs 6/7

In the light of multiple reports on the inhibitory effects of I-SMADs, inducing I-SMAD polyubiquitylation and degradation would be predicted to strongly enhance TGFβ/BMP pathway signalling. Although SMAD6/7 interact with the majority of NEDD4-like E3 ubiquitin ligases through their PY motif, these E3s primarily employ SMAD7 as an adaptor to target various substrates, including the TGFβ/BMP receptors. In the process, I-SMADs are often destroyed by proteasomal degradation [35,36]. ARKADIA, an E3 ligase that does not target the receptor complex, has been shown to target SMAD7 for ubiquitylation and degradation, thereby enhancing pathway signalling [88,89]. ARKADIA also targets multiple components of the TGFβ pathway for ubiquitylation and degradation [11,65,71,74,90–93]. However, selective SMAD7 polyubiquitylation and degradation has been reported in renal fibrosis and hypertension mouse models, causing enhanced pathway signalling [31,94].

Inhibition of I-SMAD ubiquitylation and subsequent degradation would provide a clear way to negatively control the TGFβ pathway. The histone acetyl transferase, p300, has been reported to acetylate SMAD7 at Lys64 and Lys70, the same residues in which ubiquitylation occurs. This prevents SMAD7 from being targeted by E3s for ubiquitylation and degradation [95,96]. It has also been reported that the de-acetylase SIRT1 can reverse this, creating an acetylation/de-acetylation balance controlling SMAD7 fate [97,98].

The only DUB reported to target the I-SMADs is CYLD [99]. The study performed in CYLD-knockout mice reported that CYLD targets SMAD7 protein for deubiquitylation and inhibits TGF-β signalling in the development of regulatory T cells. Moreover, CYLD appears to deubiquitylate SMAD7 at Lys360 and Lys374 but not at Lys64 or Lys70 [99].

## 11. Regulation of nuclear SMAD cofactors by reversible ubiquitylation

Once the activated R-SMAD–SMAD4 complex is translocated into the nucleus, it must then bind promoter sequences to positively or negatively regulate the expression of TGFβ response genes. However, SMAD proteins on their own have low DNA-binding affinity and require other cofactors for DNA binding [16]. Additionally, as previously described, some nuclear adaptor proteins actually inhibit SMAD–DNA binding, thereby negatively regulating SMAD transcriptional activity. Therefore, reversible ubiquitylation of nuclear co-factors can modulate TGFβ-induced transcriptional activity. RUNX2 is a transcription factor that promotes R-SMAD/DNA binding in the BMP pathway. SMURF1 has been reported to induce its ubiquitylation and degradation [100]. SMURF1 is recruited to RUNX2 by its association with SMAD6 [101]. Most other reports have concentrated on the regulation of negative nuclear cofactors SKI and SnoN that antagonize SMAD-mediated transcriptional activity. TGFβ-induced SMURF2/SMAD2 binding and targeting of SnoN release the negative regulation of SnoN on nuclear SMAD transcriptional activity in both physiological and pathological pathway signalling [33,102]. ARKADIA is reported to target both SKI and SnoN for ubiquitin-mediated degradation in a similar TGFβ-dependent fashion, leading to activation of transcriptional responses [91,92]. Later reports also identify that SKI ubiquitylation and degradation requires TGFβ signalling and ARKADIA binding to phosphorylated-SMAD2/3 [74,93]. ARKADIA function is itself regulated by binding to proteins such as AXIN and RB1CC1 [89,90]. The anaphase-promoting complex E3 ligase has also been reported to act in a similar manner by targeting SnoN [103,104], while the CDC34 E2 targets SKI and SnoN in a cell-cycle-dependent fashion [105]. Very little is known about the DUBs that reverse the ubiquitylation of the earlier-mentioned nuclear SMAD cofactors.

## 12. Concluding remarks

The TGFβ family of cytokines influences the behaviour and fate of almost every cell type in vertebrates. The cellular responses to TGFβ signals vary greatly depending on the biological context. Despite this, all cells share the fundamental transduction mechanisms of TGFβ signalling. Various post-translational modifications of key mediators of the TGFβ pathway in response to multiple signals modulate their activity, stability and subcellular localization. The integration of different signals ultimately determines the extent and duration of cellular responses to TGFβ signals. Reversible ubiquitylation of fundamental TGFβ pathway mediators offers a key regulatory balance on the outcome of the pathway. Ubiquitylation confers a versatile modification of target proteins. This versatility is further augmented by the possibility of multiple types of ubiquitin chains that can be formed on target proteins. While K48-linked polyubiquitin chains have been described to cause proteasomal degradation of TGFβ pathway components, the precise nature of polyubiquitin chains remains unexplored. Proteins that contain unique UBDs would be predicted to be essential for interpreting the signals contained within target proteins with unique polyubiquitin chains. In the TGFβ pathway, few such proteins have been identified.

Regulation of the TGFβ pathway by ubiquitylation of key components has been widely reported (table 1). While many candidate E3 ubiquitin ligases have been proposed, little is known about the E2-ubiquitin-conjugating enzymes further upstream. Several members of the NEDD4-like family of E3 ubiquitin ligases have been reported to catalyse the polyubiquitylation and degradation of both TGFβ receptors and SMAD transcription factors. Indeed, SMURF1/2 appears to be transcriptional targets of TGFβ cytokines themselves and inhibit the pathway through a negative feedback loop [18]. The observations that the recognition of SMAD1 and SMAD2/3, by SMURF1 and NEDD4L, respectively, requires phosphorylation of linker regions of SMAD proteins imply an active interplay between phosphorylation and ubiquitylation processes [57,64]. Such crosstalk is likely to happen across multiple proteins and post-translational modifications as cells respond to a constant barrage of complex extra-cellular and intra-cellular signals. The knockout mouse models of several E3 ubiquitin ligases implicated in the TGFβ pathway exist. SMURF1 knockout mice show enhanced bone mass upon ageing, phenotypes expected to result from enhanced BMP signalling [106]. While functional redundancy between SMURF1 and SMURF2 may have contributed to the lack of striking phenotypes in SMURF1- or SMURF2-knockout mice, double knockout resulted in embryonic lethality with severe defects in planar cell polarity [34,106,107]. As most E3 ubiquitin ligases implicated in the TGFβ pathway are likely to have several substrates, observed phenotypes could be attributed to effects on their most critical targets, thereby confusing any impact relating to the TGFβ pathway. Pathway-specific E3 mutants would therefore be required for such physiological studies relating to one pathway over any others targeted by E3 ligases. Understanding the molecular mechanisms by which the E3 ubiquitin ligases recognize specific substrates, and how they are activated, would be essential to producing such pathway-specific physiological mouse models. The precise mechanisms by which all reported E3 ubiquitin ligases are activated or recognize their substrates in the TGFβ pathway are still not well defined.

**Table 1.** A summary of known E3 ubiquitin ligases and deubiquitylating enzymes (DUBs) involved in TGF*β* pathway signalling. Asterisks indicate E3s also targeting tail and/or linker-phosphorylated SMAD proteins. Common alternative names for E3 ubiquitin ligases and DUBs in table: ARKADIA = ring finger 111; WWP1 = AIP5, Tiul1; NEDD4L = NEDD4-2; TRIM33 = ECTO, TIF1*γ*; ITCH = AIF4, AIP4; USP9x = FAM; UCH37 = UCHL5.

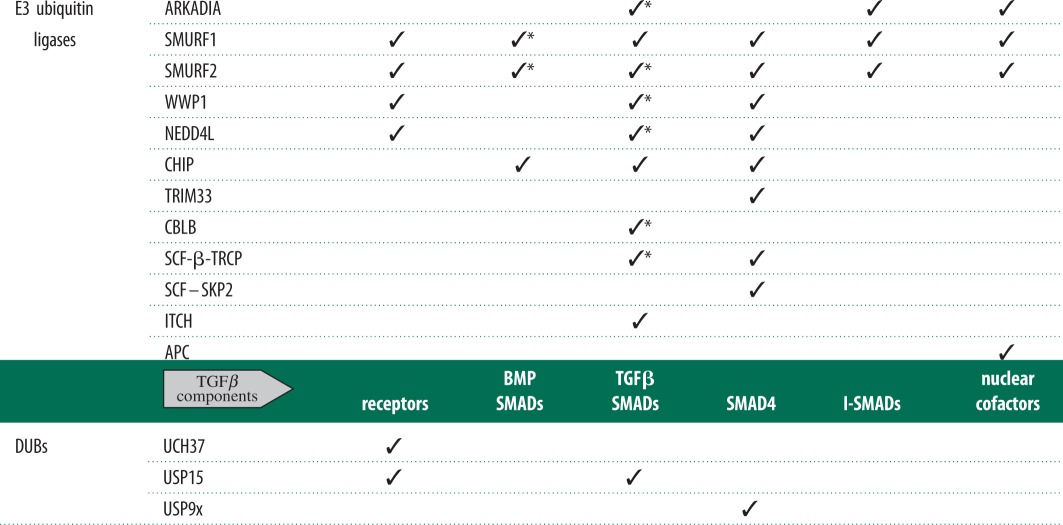

Deciphering the mechanisms of how TGFβ receptor kinases mediate the phosphorylation of R-SMADs has resulted in our understanding of the fundamental aspects of TGFβ signalling [1]. The precise ubiquitylation sites within receptors, SMAD proteins or SMAD cofactors as well as the nature of polyubiquitin chains that are attached to the initial ubiquitin are largely undefined. Most of the ubiquitylation sites reported on SMAD proteins thus far have resulted from over-expression and mutagenesis studies, which have the potential of yielding artefacts. Recent technologies capable of identifying ubiquitylated peptides on endogenous proteins hold great promise for investigating reversible ubiquitylation in the TGFβ pathway [108,109]. Indeed one of these studies was able to identify multiple ubiquitylation sites within endogenous type I TGFβ/BMP receptors as well as BMP and TGFβ ligands. That the ligands could themselves be regulated by ubiquitylation is an intriguing observation that has as yet eluded consideration entirely.

Investigation into the regulation of the TGFβ pathway by DUBs is an emerging research field. To date, only three DUBs, namely UCH37, USP9X and USP15, have been attributed a role in deubiquitylating components of the TGFβ pathway (table 1) [7,29,30,50]. The mode of substrate recognition and catalysis of reported TGFβ pathway DUBs are still undefined. Because of their limited number in the genome, DUBs are likely to be promiscuous with regard to their substrate range. Therefore, *RNAi*-based global DUB knockdown strategies employed to identify TGFβ pathway regulators have to be used cautiously. A better strategy would be to identify DUBs that associate directly with specific TGFβ pathway components. Understanding the molecular mechanisms by which DUBs recognize their substrates is critical in defining their roles on specific targets. In addition to being peptidases, the DUBs possess a characteristic in being able to recognize and bind to uniquely ubiquitylated proteins or ubiquitin chains. This ability alone, regardless of their catalytic activity, may serve an important regulatory purpose during signalling by modulating the activity, subcellular localization or stability of the target protein. Indeed, recent reports demonstrate that DUBs influence protein function independently of their deubiquitylating activity. As an example, USP7 was demonstrated to increase the binding affinity of p53 to its target genes independent of its deubiquitylase activity [110]. Similar analogies may hold true for DUBs in the TGFβ pathway.

The TGFβ pathway components are frequently compromised in numerous diseases, including fibrosis, cancer progression and metastasis [7,8,11,12,31–34,94]. Therefore, understanding the molecular mechanisms by which reversible ubiquitylation regulates TGFβ signalling may hold some therapeutic promise against these diseases. Amplification of several members of the NEDD4-like E3 ligases, including SMURF1/2, is reported to be associated with tumour progression [44]. Reduced ARKADIA activity is associated with the pathogenesis of colorectal cancers [11]. The efficacy of the proteasome inhibitor Bortezomib against B cell lymphoma demonstrates that ubiquitin ligases and the ubiquitylation system could be exploited as targets for anti-cancer therapies [111]. DUBs, which constitute the largest family of peptidases, are also associated with many human diseases, including cancer and could make attractive therapeutic targets [7,111,112]. Therefore, targeting the TGFβ-pathway-specific E2-ubiquitin-conjugating enzymes, E3-ubiquitin ligases or DUBs for inhibition may provide opportunities for the development of therapies against diseases in which the TGFβ pathway is compromised.
